# Validation of a Gene Expression Approach for the Cytological Diagnosis of Epithelioid and Biphasic Pleural Mesothelioma on a Consecutive Series

**DOI:** 10.3390/cancers15235534

**Published:** 2023-11-22

**Authors:** Rossella Bruno, Anello Marcello Poma, Greta Alì, Agnese Proietti, Alessandro Ribechini, Antonio Chella, Gabriella Fontanini

**Affiliations:** 1 Unit of Pathological Anatomy, University Hospital of Pisa, Via Roma 67, 56126 Pisa, Italy; agneseproietti@gmail.com; 2 Department of Surgical, Medical, Molecular Pathology and Critical Area, University of Pisa, Via Savi 10, 56126 Pisa, Italy; marcellopoma@gmail.com (A.M.P.); greta.ali@gmail.com (G.A.); gabriella.fontanini@unipi.it (G.F.); 3 Endoscopic Section of Pneumology, University Hospital of Pisa, Via Paradisa 2, 56124 Pisa, Italy; a.ribechini@ao-pisa.toscana.it; 4 Unit of Pneumology, University Hospital of Pisa, Via Paradisa 2, 56124 Pisa, Italy; anto.kell@tiscali.it

**Keywords:** pleural mesothelioma, mesothelial hyperplasia, pleural effusions, subtyping, gene expression

## Abstract

**Simple Summary:**

Pleural effusions are common clinical manifestations of pleural mesothelioma (PM) and often constitute the only available biological material for diagnosis. However, the cytological diagnosis of PM can be challenging. Over the years, ancillary tests, mainly based on the analysis of single biomarkers, have been developed to differentiate malignant from benign effusions, but their sensitivity is limited. In this study, we validated, on a consecutive series, a previously defined 117-gene expression panel as a diagnostic tool for the cytological diagnosis of PM. This gene panel proved to be useful not only in the differential diagnosis of PM and mesothelial hyperplasia but also in the discrimination of the two most common PM subtypes (epithelioid and biphasic) on cytology. Once the malignancy is assessed, the PM subtype definition strongly impacts therapy and prognosis. In this context, the 117-gene panel could be a powerful diagnostic tool to improve the clinical management of PM patients.

**Abstract:**

Cytological diagnosis of pleural mesothelioma (PM) is controversial, even using ancillary markers (BAP1, MTAP and CDKN2A). Here, we aimed to prospectively validate a previously developed 117-gene expression panel for the differential cytological diagnosis of epithelioid, biphasic PM and mesothelial hyperplasia. Seventy-seven pleural effusions were classified using the 117-gene expression levels (NanoString system). Sixty-eight cases were also screened for ancillary markers. The performance of both gene panel and ancillary markers was evaluated using ROC metrics. A score using the top consistently deregulated genes between epithelioid and biphasic PM was built to subtype malignant effusions. The panel alone reached a diagnostic accuracy (0.89) comparable to the best marker combination (BAP1 plus MTAP: 0.88). Ancillary tests missed 8 PMs, 7 of which were correctly classified by the panel. The score built by averaging the expression levels of *MSLN*, *CLDN15* and *CFB* showed an accuracy of 0.80 in subtyping epithelioid and biphasic effusions. The 117-gene panel is effective for PM cytological diagnosis of epithelioid and biphasic PM. This tool can be complementary to ancillary markers, reducing invasive procedures and allowing an earlier diagnosis. Finally, the possibility to subtype PM on effusions strengthens the panel’s role in PM diagnosis and management.

## 1. Introduction

Pleural mesothelioma (PM) is a rare and severe tumor affecting the pleura, strictly correlated with asbestos exposure [1]. PM usually has a lag time equal to 30–40 years between asbestos exposure and the first clinical manifestations, with limited therapeutic options and overall survival (OS) ranging from 4 to 14 months [2,3]. There are three main PM histotypes—epithelioid (70–80% of cases), biphasic (10–20%), and sarcomatoid (10%) [4,5,6]—with different therapeutic and prognostic implications. In fact, epithelioid PM patients are potentially eligible for surgery and a multimodality approach, and have a better OS compared to other histotypes [2,7,8].

The diagnosis of PM can be extremely challenging both for small tissue biopsies if tissue invasion is not clearly assessable and for pleural effusions [9,10]. In particular, the morphologic differentiation of PM from reactive mesothelial hyperplasia (MH) is not always possible for effusions so a tissue sample can be required first to confirm malignancy and then to subtype PM [5,11]. According to the International Mesothelioma Interest Group (IMIG) recommendations, a cytological diagnosis of PM is possible by coupling morphological examination with ancillary tests [12,13]. BRCA1-associated protein 1 (BAP1) and cyclin-dependent kinase inhibitor 2A (*CDKN2A*, better known as *p16*) are the most valuable markers for the differential diagnosis of malignant and benign pleural lesions [14]. Loss of the expression of BAP1, evaluated by immunohistochemistry (IHC), and the presence of *p16* homozygous deletion evaluated by Fluorescent In Situ Hybridization (FISH), are highly specific markers for PM, since these alterations have never been described in benign lesions [14,15,16,17,18]. Furthermore, methylthioadenosine phosphorylase (MTAP) has been recently suggested as a valuable surrogate marker for *p16*, more easily evaluable by IHC rather than by FISH. Indeed, *MTAP* and *CDKN2A* genes map closely on the same chromosome (9p21), and if a deletion is present both genes are usually involved. A high concordance has been reported between MTAP loss of expression and *p16* deletion [19]. Taken separately, BAP1, *p16* and MTAP have a low diagnostic sensitivity (40–60%) for PM, which can be greatly improved by their combination (70–90%) [2,5,13,18]. However, the absence of BAP1, *p16* or MTAP alterations does not rule out the possibility of PM [2,5,14]. In addition, the cytological diagnosis of biphasic PM is even more challenging than that of epithelioid PM, considering that cells from the sarcomatoid component do not shed into the effusions [14] and the efficacy of available markers has not been completely investigated for this subtype [4,9,20].

In this context, our group previously developed a 117-gene expression panel and a related classification model able to discriminate epithelioid PM from MH [21]. This panel has already been tested and successfully compared with BAP1 and *p16* on two independent retrospective series: one including malignant (epithelioid PM) and benign pleural tissues, giving a sensitivity of 97% and a specificity of 100% [22], and the other including only pleural effusions (both cell blocks and stained smears) with a sensitivity and specificity both equal to 100% [23]. We have already demonstrated that among the 117 genes included in the panel, there are some histotype-specific markers whose expression can help discriminate between biphasic and epithelioid PM [24].

In this study, we performed a prospective validation of the 117-gene expression panel as a diagnostic tool for PM cytological diagnosis and we compared its performance with that of BAP1, p16 and MTAP. Our purpose was to improve the diagnostic algorithm for the cytological differential diagnosis not only of epithelioid PM and MH, but also of biphasic PM.

## 2. Materials and Methods

### 2.1. Study Population

From 2019 to 2021, pleural effusions from patients consecutively diagnosed with epithelioid, biphasic PM and MH were collected and evaluated at our institution (Unit of Pathological Anatomy, University Hospital of Pisa). This study was conducted in accordance with the principles of the 1975 Helsinki Declaration and was approved by the local Ethics Committee. Molecular analyses required for this study did not interfere with routine clinical practice. A written informed consent for diagnostic procedures and molecular tests was obtained from patients at the time of sampling. Informed written consent for publication was not required because all cases were completely anonymous and no sensitive data able to identify patients were used.

Both cell blocks and Papanicolaou-stained smears were collected for this study. For cell blocks, hematoxylin- and eosin-stained sections were prepared for pathological diagnosis, whereas all cytologic smears were fixed in Cytofix (Kaltek s.r.l., Padua, Italy) and stained with Papanicolaou, according to our standard laboratory protocol [23]. Histological confirmation of diagnosis was available for all PM cases, while sufficient follow-up clinical data were collected to exclude primitive or secondary malignancy (more than 20 months of follow-up) for MH cases.

BAP1, *p16*, MTAP and gene expression tests were blindly performed. Cytological and histological diagnoses were independently reviewed by three expert pathologists (G.A., A.P. and G.F.) according to the 2021 World Health Organization criteria [4] and discordant cases were discussed until an agreement was reached.

### 2.2. Gene Expression Analysis

The custom gene expression panel included 117 target genes, specifically deregulated in PM, and 6 housekeeping genes [21]. The gene expression analysis was performed on mRNA from pleural effusions by using the nCounter NanoString system (NanoString Technologies, Seattle, WA, USA), as previously described [21,23].

The percentage of atypical mesothelial cells was estimated for each case (10% was the lowest acceptable value), and a tissue enrichment was performed by manual macro-dissection before RNA purification. In detail, for cytologic smears, one stained slide was placed in xylene for 48 h to remove the coverslip; then, the slide was rehydrated in a graded ethanol series (99%, 95%, 70%, and 50%) for 10 min each [23]. Instead, two to three unstained sections (5 µm thick) underwent standard deparaffinization for each cell block. RNA was purified using the Qiagen RNeasy FFPE kit (Qiagen, Hilden, Germany), according to the manufacturer’s instructions. A total of 150 ng of RNA, assessed by an Xpose spectrophotometer (Trinean, Gentbrugge, Belgium), was hybridized with capture and reporter probes in each reaction. Hybridization was performed for 18 h at 65 °C in a SensoQuest thermal cycler (SensoQuest, Gottingen, Germany). Cleanup of the samples and the counts of digital reports were performed as described by the manufacturer (NanoString Technologies).

### 2.3. BAP1, MTAP, p16 Tests

BAP1 and MTAP IHC were conducted on cell blocks by using a mouse monoclonal anti-BAP1 antibody (C-4; Santa Cruz Biotechnology, Inc., Dallas, TX, USA) at 1:100 dilution and rabbit monoclonal anti-MTAP antibody (EPR6893; Abcam, Cambridge, UK) at 1:500 dilution, with the OptiView DAB IHC Detection Kit and OptiView Amplification Kit (Ventana Medical Systems, Tucson, AZ, USA), according to the manufacturer’s protocol. Immunostaining was executed on a BenchMark XT automated slide stainer (Ventana Medical Systems).

**Figure 1:**
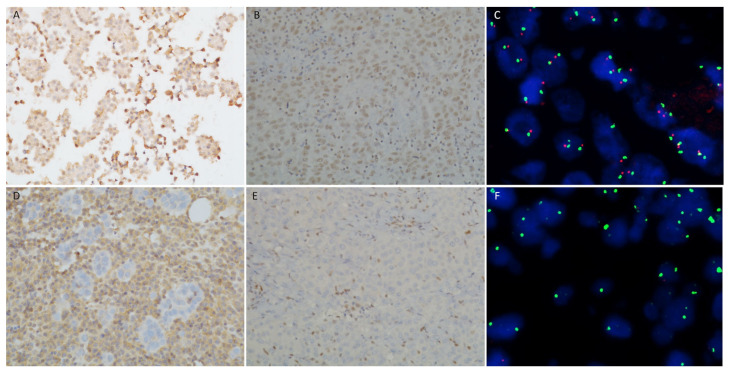
Ancillary tests for mesothelioma differential diagnosis. (**A**) Epithelioid pleural mesothelioma retaining BAP1 expression (magnification 10×). (**B**) Epithelioid pleural mesothelioma retaining MTAP expression (magnification 10×). (**C**) Epithelioid pleural mesothelioma without p16 deletion (magnification 10×). (**D**) Epithelioid pleural mesothelioma with BAP1 loss of expression (magnification 10×). (**E**) Epithelioid pleural mesothelioma with MTAP loss of expression (magnification 60×). (**F**) Epithelioid pleural mesothelioma with p16 deletion (magnification 60×).

The absence of BAP1 nuclear staining in all the atypical mesothelial cells in the presence of a positive internal control defined a case as positive for BAP1 loss (Figure 1A,D).

The absence of MTAP cytoplasmatic staining in all the atypical mesothelial cells in the presence of a positive internal control defined a case as positive for MTAP loss (Figure 1B,E).

To evaluate *p16* homozygous deletion, FISH was used with the commercially available probe Vysis LSI CDKN2A (p16) spectrum orange/CEP 9 spectrum green kit (Abbott Laboratories, Des Plaines, IL, USA), following the manufacturer’s instructions. A minimum of 60 cells were analyzed in each case. A *p16* homozygous deletion pattern observed in more than 11% of atypical mesothelial cells defined a case as positive for this alteration (Figure 1C,F).

Only cell blocks underwent BAP1, *p16* and MTAP tests. BAP1 and MTAP IHC assays are optimized for formalin-fixed, paraffin-embedded (FFPE) slides; *p16* FISH was not executed for stained smears, because only two smears were available for each case: one had to be preserved for legal purposes, the other was used for gene expression testing.

### 2.4. Data Analysis

Gene expression counts were normalized as previously reported by using the nSolver software (version 4.0) (NanoString Technologies, Seattle, WA, USA)) [23]. Normalized counts were log2-transformed for downstream analyses. Principal component analysis (PCA) was performed following the procedures of PCA tools v.10.0 package and using the entire set of genes to highlight gross patterns of expression. Samples were then grouped by unsupervised clustering based on Euclidean distance and using the Ward method implemented in the heatmap3 v.1.1.9 package. Gene expression levels of pleural tissues from our previous study [21] were employed as training sets. In detail, a random forest algorithm with logistic regression as a node model was used following the procedures of the caret v.6.0-94 package. The normalized counts produced in this study were used as a test set, and the model performance was assessed using receiver operating characteristics (ROC) metrics. Performance of the BAP1, *p16* and MTAP tests and the gene panel in cases with complete analyses (*n* = 65) were evaluated by ROC metrics using the pROC v.1.18.0 package; for the gene panel, class predictions were used in the analysis. Confidence intervals (CI) were estimated by 2000 bootstrap resampling. Differential expression analysis was conducted by moderated t-statistics, following the procedures of the limma v.3.54.2 package. The score to distinguish epithelial from biphasic PM was built by averaging the expression levels of the top 3 consistently deregulated genes in tissues and effusions. All analyses and plots were generated in R environment (v.4.2.2, https://www.r-project.org/, last accessed 13 February 2023).

## 3. Results

### 3.1. Study Population

In this study, a total of 85 patients with PM or MH were consecutively enrolled. In detail, the epithelioid PM cases were 36 (27 cell blocks and 9 Papanicolaou-stained smears; 10 females and 26 males, with a median age of 71 years); biphasic PM were 15 (12 cell blocks and 3 Papanicolaou-stained smears; 2 females and 13 males, with a median age of 71 years); MH were 34 (33 cell blocks and 1 Papanicolaou-stained smear; 17 females and 17 males, with a median age of 70 years). Eight samples (3 Papanicolaou stained smears, including 1 MH and 2 epithelioid PMs, and 5 cell blocks, including 1 MH and 4 epithelioid PM cases) were not adequate (less than 10% of atypical cells, poor quantity and quality RNA or consumed cell blocks) neither for IHC, FISH, nor for gene expression analyses.

Finally, 77 cases successfully underwent gene expression analysis, 68 were cell blocks and were also screened for BAP1, MTAP and *p16*.

### 3.2. Gene Expression Analysis

All 77 samples passed the quality checks and were considered adequate for statistical and bioinformatics analysis.

**Figure 2:**
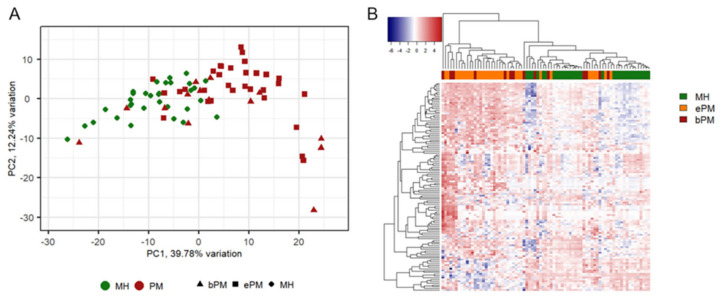
Principal component analysis and unsupervised clustering. (**A**) Principal component analysis was performed on normalized log2 counts. A degree of separation between MH and PM can be observed on principal component 1. (**B**) Unsupervised clustering of samples using scaled and centered counts was performed based on Euclidean distance and using Ward’s method. Two main clusters were produced: one specific for malignant cases and the other enriched with benign pleural effusions. Red and blue indicate a high and low gene expression level, respectively. Each column represents a single sample, and each row represents a single gene. MH, reactive mesothelial hyperplasia; PM, pleural mesothelioma; ePM, epithelioid pleural mesothelioma; bPM, biphasic pleural mesothelioma.

After PCA, the first two principal components (PC) accounted for more than 50% of the variability and were used for plotting. MH and PM were separated on PC1 with some overlapping (Figure 2A). Unsupervised clustering revealed different gene expression profiles for malignant and benign cases; in particular, it is possible to identify two main clusters, one including only malignant and one including mainly benign cases (Figure 2B).

Considering all the 77 samples (both cell blocks and stained smears), the classification model had an area under the curve (AUC) equal to 0.90 (95% CI 0.81–0.97), a sensitivity of 0.84 (95% CI 0.71–0.93), a specificity of 1 (95% CI 0.94–1), an accuracy of 0.91 (95% CI 0.83–0.96), a positive predictive value (PPV) of 1 (95% CI 0.95–1), and a negative predictive value (NPV) of 0.82 (95% CI 0.71–0.91). Moreover, it showed similar performance in the differential diagnosis of both epithelioid (27 out of 30 cases correctly classified as malignant) and biphasic PM (13 out of 15 cases correctly classified) from benign effusions.

Deregulated genes between epithelioid and biphasic PM effusions were determined and compared with the already reported deregulated genes between epithelioid and biphasic PM pleural tissues [24]. The top consistently deregulated genes between epithelioid and biphasic PM cases—both on pleural tissues and effusions—were *MSLN*, *CLDN15* and *CFB* (Supplementary Table S1). The score built by averaging the expression levels of these genes showed an accuracy of 0.80 (95% CI 0.63–0.90) in discriminating epithelioid and biphasic PM directly on pleural effusions.

### 3.3. BAP1, MTAP, p16 Tests

BAP1 IHC was successfully executed on all 68 cell blocks; MTAP IHC provided assessable results in 66 out of 68 cases, while *p16* FISH was in 65 out of 68 cases. In detail, two cases (both MHs) were indeterminate for MTAP because of inadequate staining in internal control positive cells and/or heterogeneous staining in mesothelial cells. Three cases (1 epithelioid PM and 2 biphasic PMs) were not evaluable for *p16*, probably due to their suboptimal pre-analytical conditions (i.e., fixation time, storage).

**Figure 3:**
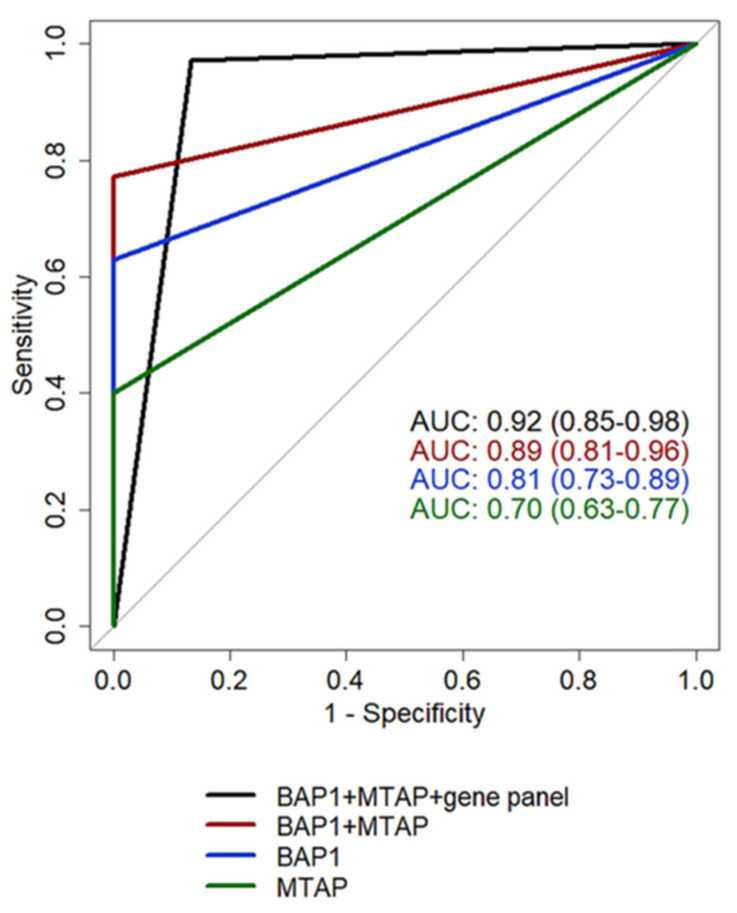
ROC curves: ROC curves of ancillary tests alone or in combination with the 117-gene panel. AUC, area under the curve.

Overall, 65 cases provided clear results for BAP1, MTAP, *p16* and for the 117-gene expression panel. These cases were used to compare the performance of single biomarkers and their combinations, as reported in Table 1 and Figure 3. Interestingly, 8 PM cases showed neither BAP1 nor MTAP/*p16* alterations. Seven out of 8 misdiagnosed PM cases were correctly classified as malignant using the 117-gene expression panel.

In addition, we compared gene expression levels of *BAP1* and *CDKN2A* included in our gene panel with IHC expression of BAP1 and the presence of *CDKN2A* deletion evaluated by FISH (Supplementary Figures S1 and S2), and no strong agreement was found.

## 4. Discussion

Pleural effusions are among the first clinical manifestations of PM and can provide important diagnostic information [4,11,13,14]. However, the diagnosis of PM on pleural effusions is still a controversial issue and several factors must be taken into account: cytomorphologic features overlap between PM and MH cells, and this fact, along with the lack of standardization [9] in the collection and preparation procedures, can result in variable interpretations across cytopathologists. The introduction of BAP1, MTAP and *p16* tests has greatly improved the diagnostic accuracy of pleural effusions, but it is necessary to perform at least two of these tests (i.e., BAP1 and MTAP) to reach a good sensitivity [4,5,14,17,25,26]. Although theoretically evaluable both on cell blocks and smears, IHC tests are standardized mainly on cell blocks, and stained smears are not always sufficient to perform more than one molecular test. In addition, interpretation challenges are well-known (i.e., imperfect internal control staining, a mix of nuclear and cytoplasmic staining), and heterogeneous staining is frequently observed in overlapping cells, typical of cytology samples [19]. BAP1 and MTAP IHC have been reported to provide ambiguous results in 5% of tissue samples, and this rate is equal to 10% for cell blocks [5,19,27]. In these cases, it is necessary to perform a biopsy, which delays the start of treatment. Moreover, not all patients tolerate invasive diagnostic procedures [9,10].

In this study, we validated the 117-gene panel as a diagnostic tool for the cytological diagnosis of PM: as a single test, this panel reached a sensitivity and a diagnostic accuracy comparable to those of combined BAP1 and MTAP evaluation. Data interpretation is more objective and the analysis can be executed directly on stained smears, just after pathological evaluation, thus avoiding cell block preparation and saving time. It has been reported that when a cytological diagnosis is reliable and treatment can be immediately initiated median OS can be extended [5,28]. Moreover, the nCounter NanoString procedure is based on direct counting of the mRNA target molecules, with no amplification step [28,29], so that RNA quantity and quality, usually scarce from FFPE or stained smears, are not limiting factors and the failure rate is extremely low [30]. In our study, none of the analyzed 77 cases failed the gene expression analysis.

In this prospective cohort, we also confirmed the usefulness of BAP1 and MTAP IHC tests on pleural effusions, whose combination reached excellent diagnostic accuracy. On the contrary, in our series, the *p16* FISH test did not improve diagnostic performance. However, 8 out of 35 PM cell blocks (22.8%) would not have been correctly diagnosed by ancillary tests. Seven out of eight were correctly classified as malignant by the NanoString panel, thus reducing the rate of false negative cases to 2.8% and increasing both diagnostic accuracy and sensitivity.

A limit of this approach is that specificity may be slightly reduced, since the gene panel misclassified some MH samples. Consistently with our previous studies, we applied the same classification algorithm that uses all 117 genes. There is still room for improvement both in terms of gene selection and testing of different algorithms.

We are aware that not all routine laboratories are equipped with a NanoString system and IHC is a more affordable first-level test. However, gene expression analysis can be considered before planning a biopsy in non-decisive pleural fluids, possibly referring to specialized centers.

Another advantage of the 117-gene panel is the possibility to evaluate more genes simultaneously and to obtain much more information alongside the differential diagnosis between malignant and benign pleural effusions. PM subtyping drives patients’ therapy and prognosis definition, but biphasic PM can be difficult to diagnose and discriminate from other histotypes [31]. It is composed of both epithelioid and sarcomatoid morphology [4,9], and the higher the proportion of the sarcomatoid component, the worse the prognosis [31]. However, the identification of a spindle cell component is not easy and a considerable interobserver variation in recognizing biphasic subtypes has been reported [31,32]. On pleural effusions it is impossible to morphologically discriminate between biphasic and epithelioid PMs, considering that sarcomatoid cells do not shed in the effusions [14]. It has already been demonstrated that gene expression profiles on tumor tissues can improve PM subtyping, particularly for biphasic PM [33,34]; however, to the best of our knowledge, no data are available concerning the role of gene expression profiles in PM subtyping on pleural effusions. In a previous study, performed on a retrospective series of pleural tissues from patients with epithelioid, biphasic and sarcomatoid PM, we found that our panel includes some genes expressed in a histotype-specific manner [24]. In this work, we confirmed the deregulation of *CFB*, *MSLN* and *CLDN15* on pleural effusions from epithelioid and biphasic PM. The score built by averaging the expression levels of these genes showed an accuracy of 0.80 in discriminating cytological specimens from the two most common histotypes. In this context, once malignancy has been assessed, the possibility of subtyping epithelioid and biphasic PM directly on pleural effusions can suggest another important clinical application of this gene expression panel.

It has to be acknowledged that the malignant rate of our study is probably higher than most consecutive series. This can be due both to the lack of sufficient material from some benign effusions and to the elevated number of patients with suspicion of malignancy referring to our institution.

Finally, pleural effusions are precious diagnostic material for mesothelioma and the 117-gene panel could improve the clinical management of PM patients both as a single test or in a stepwise diagnostic approach.

## 5. Conclusions

The implementation of cytological diagnosis of PM is warranted in order to provide patients with an earlier diagnosis reducing the number of invasive procedures. This study confirmed the effectiveness of the 117-gene panel for the cytological differential diagnosis of both epithelioid and biphasic PM from benign effusions. Furthermore, the use of gene expression profiles to subtype PM directly on pleural effusions strengthens its diagnostic role.

## Figures and Tables

**Table 1:** Performance of tested biomarkers calculated on 65 pleural effusions analyzed by all methods. CI, confidence interval; AUC, area under the curve; PPV, positive predictive value; NPV, negative predictive value. * computed on a two-step approach: first, BAP1 and MTAP; then, the result of the 117-gene panel was considered on all cases that retained the BAP1 and MTAP expressions.

	BAP1	p16	MTAP	BAP1 + MTAP	117 Gene Panel	BAP1 + MTAP + 117 Gene Panel *
**AUC** **(95%CI) **	0.81 (0.73–0.89)	0.68 (0.61–0.77)	0.70 (0.63–0.77)	0.89 (0.81–0.96)	0.87 (0.76–0.96)	0.92 (0.85–0.98)
**Sensitivity** **(95%CI) **	0.63 (0.46–0.77)	0.36 (0.21–0.52)	0.40 (0.23–0.57)	0.77 (0.63–0.89)	0.80 (0.69–0.94)	0.97 (0.91–1)
**Specificity** **(95%CI) **	1 (1–1)	1 (1–1)	1 (1–1)	1 (1–1)	1 (0.93–1)	0.87 (0.73–0.97)
**Accuracy** **(95%CI) **	0.80 (0.71–0.88)	0.67 (0.59–0.75)	0.68 (0.58–0.77)	0.88 (0.80–0.94)	0.89 (0.83–0.95)	0.92 (0.86–0.98)
**PPV** **(95%CI) **	1 (1–1)	1 (1–1)	1 (1–1)	1 (1–1)	1 (0.94–1)	0.90 (0.81–0.97)
**NPV** **(95%CI)**	0.70 (0.61–0.79)	0.59 (0.54–0.65)	0.59 (0.53–0.67)	0.79 (0.70–0.88)	0.81 (0.73–0.94)	0.96 (0.89–1)

## Data Availability

Data supporting the findings of this study is available from the authors on reasonable request.

## Supplementary Materials

The following supporting information can be downloaded at: https://www.mdpi.com/article/10.3390/cancers15235534/s1, Table S1: Gene expression comparison between pleural effusions from biphasic versus epithelioid mesothelioma. Figure S1: Comparison of BAP1 mRNA and protein expression levels, evaluated by nCounter NanoString system and immunohistochemistry, respectively. Figure S2: Comparison of *CDKN2A* mRNA levels and presence of gene deletion, evaluated by nCounter NanoString system and fluorescence in situ hybridization, respectively.

## References

[1] Robinson B.W.S., Musk A.W., Lake R.A. (2005). Malignant Mesothelioma. Lancet.

[2] Popat S., Baas P., Faivre-Finn C., Girard N., Nicholson A.G., Nowak A.K., Opitz I., Scherpereel A., Reck M. (2022). Malignant Pleural Mesothelioma: ESMO Clinical Practice Guidelines for Diagnosis, Treatment and Follow-Up. Ann. Oncol..

[3] Robinson B.W.S., Lake R.A. (2005). Advances in Malignant Mesothelioma. N. Engl. J. Med..

[4] Sauter J.L., Dacic S., Galateau-Salle F., Attanoos R.L., Butnor K.J., Churg A., Husain A.N., Kadota K., Khoor A., Nicholson A.G. (2022). The 2021 WHO Classification of Tumors of the Pleura: Advances Since the 2015 Classification. J. Thorac. Oncol..

[5] Nabeshima K., Hamasaki M., Kinoshita Y., Matsumoto S., Sa-ngiamwibool P. (2022). Update of Pathological Diagnosis of Pleural Mesothelioma Using Genomic-based Morphological Techniques, for Both Histological and Cytological Investigations. Pathol. Int..

[6] Savic I., Myers J. (2021). Update on Diagnosing and Reporting Malignant Pleural Mesothelioma. Acta Med. Acad..

[7] Opitz I. (2014). Management of Malignant Pleural Mesothelioma-The European Experience. J. Thorac. Dis..

[8] Brcic L., Kern I. (2020). Clinical Significance of Histologic Subtyping of Malignant Pleural Mesothelioma. Transl. Lung Cancer Res..

[9] Beasley M.B., Galateau-Salle F., Dacic S. (2021). Pleural Mesothelioma Classification Update. Virchows Arch..

[10] Dacic S. (2022). Pleural Mesothelioma Classification—Update and Challenges. Mod. Pathol..

[11] Hjerpe A., Ascoli V., Bedrossian C., Boon M., Creaney J., Davidson B., Dejmek A., Dobra K., Fassina A., Field A. (2015). Guidelines for Cytopathologic Diagnosis of Epithelioid and Mixed Type Malignant Mesothelioma. Complementary Statement from the International Mesothelioma Interest Group, Also Endorsed by the International Academy of Cytology and the Papanicolaou Society of Cytopathology. CytoJournal.

[12] Churg A., Nabeshima K., Ali G., Bruno R., Fernandez-Cuesta L., Galateau-Salle F. (2018). Highlights of the 14th International Mesothelioma Interest Group Meeting: Pathologic Separation of Benign from Malignant Mesothelial Proliferations and Histologic/Molecular Analysis of Malignant Mesothelioma Subtypes. Lung Cancer.

[13] Klebe S., Galateau Salle F., Bruno R., Brcic L., Chen-Yost H.I., Jaurand M.-C. (2022). The Highlights of the 15th International Conference of the International Mesothelioma Interest Group—Do Molecular Concepts Challenge the Traditional Approach to Pathological Mesothelioma Diagnosis?. Lung Cancer.

[14] Monaco S.E., Brcic L., Dacic S. (2022). State-of-the-Art Cytology of Pleural Fluid, Focusing on the Diagnosis of Mesothelioma. Cytopathol. Off. J. Br. Soc. Clin. Cytol..

[15] Chevrier M., Monaco S.E., Jerome J.A., Galateau-Salle F., Churg A., Dacic S. (2020). Testing for BAP1 Loss and CDKN2A/P16 Homozygous Deletion Improves the Accurate Diagnosis of Mesothelial Proliferations in Effusion Cytology. Cancer Cytopathol..

[16] Hwang H.C., Sheffield B.S., Rodriguez S., Thompson K., Tse C.H., Gown A.M., Churg A. (2016). Utility of BAP1 Immunohistochemistry and P16 (CDKN2A) FISH in the Diagnosis of Malignant Mesothelioma in Effusion Cytology Specimens. Am. J. Surg. Pathol..

[17] Walts A.E., Hiroshima K., McGregor S.M., Wu D., Husain A.N., Marchevsky A.M. (2016). BAP1 Immunostain and CDKN2A (P16) FISH Analysis: Clinical Applicability for the Diagnosis of Malignant Mesothelioma in Effusions. Diagn. Cytopathol..

[18] Berg K.B., Dacic S., Miller C., Cheung S., Churg A. (2018). Utility of Methylthioadenosine Phosphorylase Compared with BAP1 Immunohistochemistry, and CDKN2A and NF2 Fluorescence In Situ Hybridization in Separating Reactive Mesothelial Proliferations from Epithelioid Malignant Mesotheliomas. Arch. Pathol. Lab. Med..

[19] Hamasaki M., Kinoshita Y., Yoshimura M., Matsumoto S., Kamei T., Hiroshima K., Sato A., Tsujimura T., Kawahara K., Nabeshima K. (2019). Cytoplasmic MTAP Expression Loss Detected by Immunohistochemistry Correlates with 9p21 Homozygous Deletion Detected by FISH in Pleural Effusion Cytology of Mesothelioma. Histopathology.

[20] Lynggård L.A., Panou V., Szejniuk W., Røe O.D., Meristoudis C. (2022). Diagnostic Capacity of BAP1 and MTAP in Cytology from Effusions and Biopsy in Mesothelioma. J. Am. Soc. Cytopathol..

[21] Bruno R., Alì G., Giannini R., Proietti A., Lucchi M., Chella A., Melfi F., Mussi A., Fontanini G. (2017). Malignant Pleural Mesothelioma and Mesothelial Hyperplasia: A New Molecular Tool for the Differential Diagnosis. Oncotarget.

[22] Alì G., Bruno R., Poma A., Proietti A., Ricci S., Chella A., Melfi F., Ambrogi M., Lucchi M., Fontanini G. (2019). A Gene-expression-based Test Can Outperform Bap1 and P16 Analyses in the Differential Diagnosis of Pleural Mesothelial Proliferations. Oncol. Lett..

[23] Bruno R., Alì G., Poma A.M., Proietti A., Libener R., Mariani N., Niccoli C., Chella A., Ribechini A., Grosso F. (2020). Differential Diagnosis of Malignant Pleural Mesothelioma on Cytology. J. Mol. Diagn..

[24] Bruno R., Poma A.M., Alì G., Distefano C., Proietti A., Chella A., Lucchi M., Melfi F., Franco R., Fontanini G. (2022). Gene Expression Analysis of Biphasic Pleural Mesothelioma: New Potential Diagnostic and Prognostic Markers. Diagnostics.

[25] Andrici J., Sheen A., Sioson L., Wardell K., Clarkson A., Watson N., Ahadi M.S., Farzin M., Toon C.W., Gill A.J. (2015). Loss of Expression of BAP1 Is a Useful Adjunct, Which Strongly Supports the Diagnosis of Mesothelioma in Effusion Cytology. Mod. Pathol..

[26] Cozzi I., Oprescu F.A., Rullo E., Ascoli V. (2018). Loss of BRCA1-Associated Protein 1 (BAP1) Expression Is Useful in Diagnostic Cytopathology of Malignant Mesothelioma in Effusions. Diagn. Cytopathol..

[27] Hida T., Hamasaki M., Matsumoto S., Sato A., Tsujimura T., Kawahara K., Iwasaki A., Okamoto T., Oda Y., Honda H. (2017). Immunohistochemical Detection of MTAP and BAP1 Protein Loss for Mesothelioma Diagnosis: Comparison with 9p21 FISH and BAP1 Immunohistochemistry. Lung Cancer.

[28] Geiss G.K., Bumgarner R.E., Birditt B., Dahl T., Dowidar N., Dunaway D.L., Fell H.P., Ferree S., George R.D., Grogan T. (2008). Direct Multiplexed Measurement of Gene Expression with Color-Coded Probe Pairs. Nat. Biotechnol..

[29] Tsang H.-F., Xue V.W., Koh S.-P., Chiu Y.-M., Ng L.P.-W., Wong S.-C.C. (2017). NanoString, a Novel Digital Color-Coded Barcode Technology: Current and Future Applications in Molecular Diagnostics. Expert Rev. Mol. Diagn..

[30] Rogers T.-M., Arnau G.M., Ryland G.L., Huang S., Lira M.E., Emmanuel Y., Perez O.D., Irwin D., Fellowes A.P., Wong S.Q. (2017). Multiplexed Transcriptome Analysis to Detect ALK, ROS1 and RET Rearrangements in Lung Cancer. Sci. Rep..

[31] Galateau Salle F., Le Stang N., Nicholson A.G., Pissaloux D., Churg A., Klebe S., Roggli V.L., Tazelaar H.D., Vignaud J.M., Attanoos R. (2018). New Insights on Diagnostic Reproducibility of Biphasic Mesotheliomas: A Multi-Institutional Evaluation by the International Mesothelioma Panel From the MESOPATH Reference Center. J. Thorac. Oncol..

[32] Brčić L., Jakopović M., Brčić I., Klarić V., Milošević M., Šepac A., Samaržija M., Seiwerth S. (2014). Reproducibility of Histological Subtyping of Malignant Pleural Mesothelioma. Virchows Arch..

[33] Alcala N., Mangiante L., Le-Stang N., Gustafson C.E., Boyault S., Damiola F., Alcala K., Brevet M., Thivolet-Bejui F., Blanc-Fournier C. (2019). Redefining Malignant Pleural Mesothelioma Types as a Continuum Uncovers Immune-Vascular Interactions. eBioMedicine.

[34] Bueno R., Stawiski E.W., Goldstein L.D., Durinck S., De Rienzo A., Modrusan Z., Gnad F., Nguyen T.T., Jaiswal B.S., Chirieac L.R. (2016). Comprehensive Genomic Analysis of Malignant Pleural Mesothelioma Identifies Recurrent Mutations, Gene Fusions and Splicing Alterations. Nat. Genet..

