# Supplementary material for: Validation of a Gene Expression Approach for the Cytological Diagnosis of Epithelioid and Biphasic Pleural Mesothelioma on a Consecutive Series
Source: Cancers (Basel). 2023 Nov 22;15(23):5534. doi: 10.3390/cancers15235534 (PMC10705177; doi:10.3390/cancers15235534)
Supplement: Supplementary file 1 [file cancers-15-05534-s001.zip › Supplementary Figures.pdf]

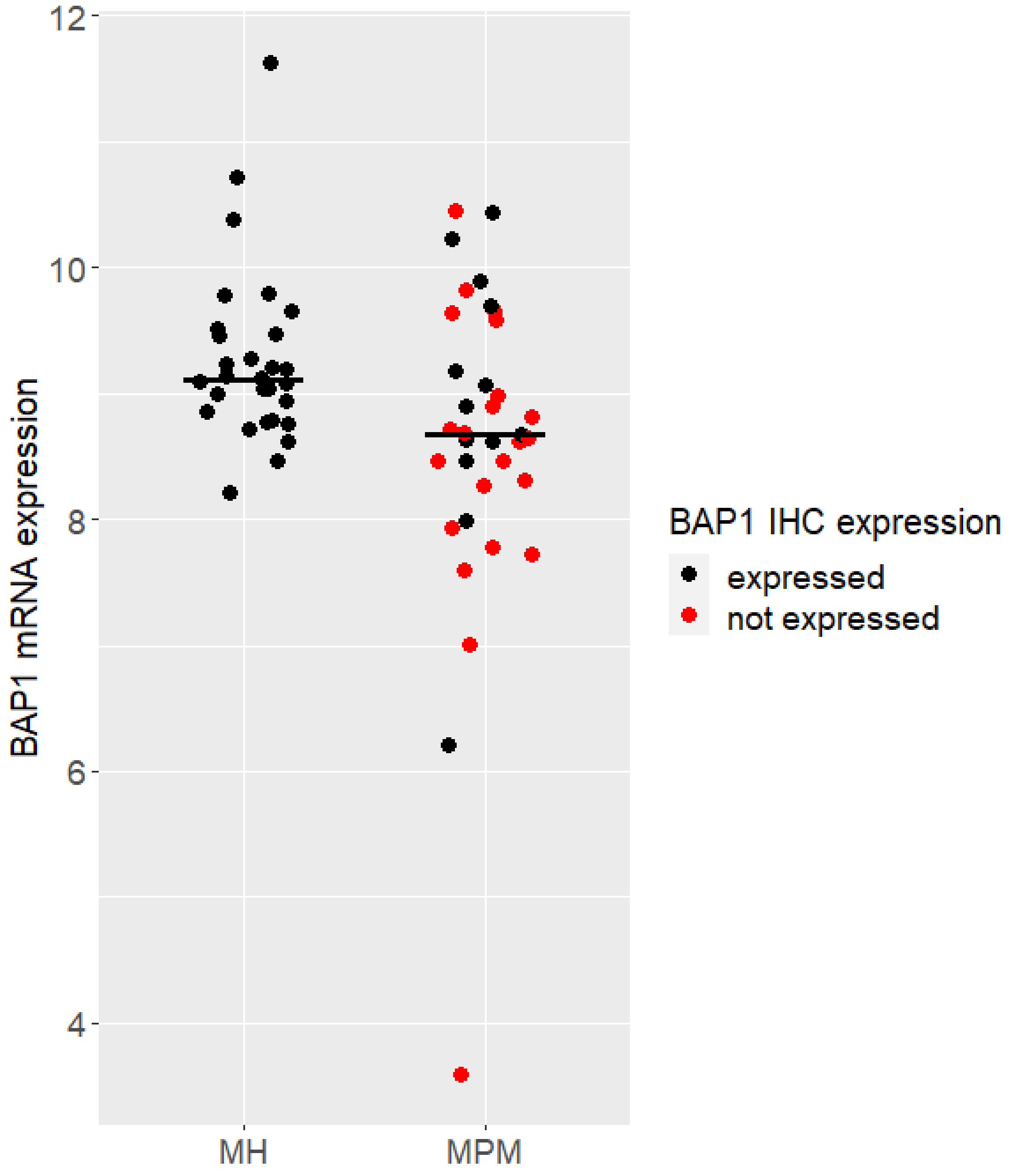

Figure S1. Comparison of BAP1 mRNA and protein expression levels, evaluated by nCounter NanoString system and immunohistochemistry, respectively.

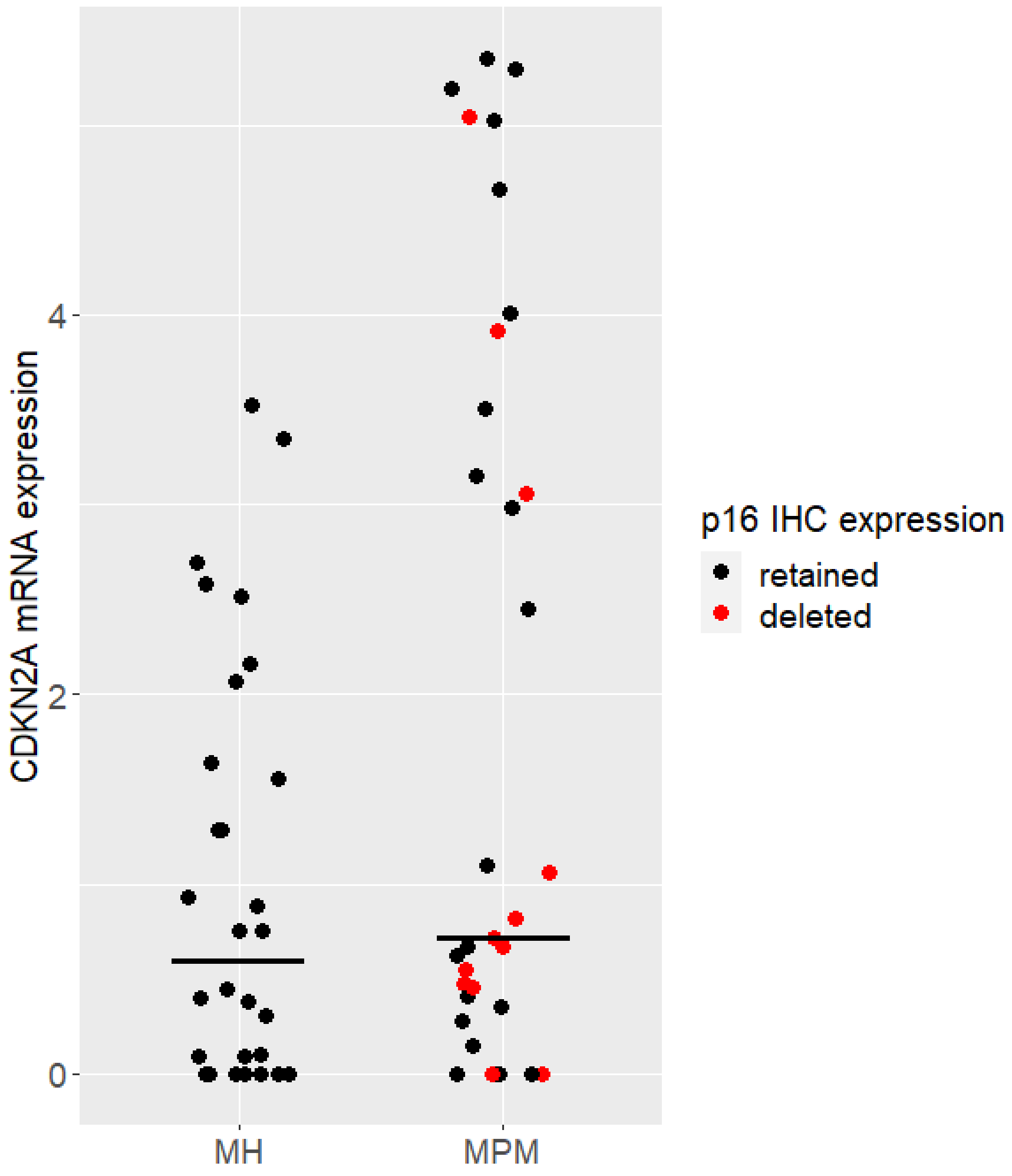

Figure S2. Comparison of *CDKN2A* mRNA levels and presence of gene deletion, evaluated by nCounter NanoString system and fluorescence in situ hybridization, respectively.
